# Fluorometric determination of ethidium bromide efflux kinetics in *Escherichia coli*

**DOI:** 10.1186/1754-1611-3-18

**Published:** 2009-10-16

**Authors:** Laura Paixão, Liliana Rodrigues, Isabel Couto, Marta Martins, Pedro Fernandes, Carla CCR de Carvalho, Gabriel A Monteiro, Filipe Sansonetty, Leonard Amaral, Miguel Viveiros

**Affiliations:** 1 Unit of Mycobacteriology, Instituto de Higiene e Medicina Tropical, Universidade Nova de Lisboa (IHMT/UNL), Rua da Junqueira 100, 1349-008 Lisboa, Portugal; 2 UPMM, IHMT/UNL, Rua da Junqueira 100, 1349-008 Lisboa, Portugal; 3 Centro de Recursos Microbiológicos (CREM), Faculdade de Ciências e Tecnologia, UNL, 2829-516 Caparica, Portugal; 4 IBB - Institute for Biotechnology and Bioengineering, Centre for Biological and Chemical Engineering, Instituto Superior Técnico, Av Rovisco Pais, 1049-001 Lisboa, Portugal; 5 Instituto de Patologia e Imunologia Molecular, Universidade do Porto, Rua Dr Roberto Frias, 4200-465 Porto, Portugal; 6 Cost Action BM0701 (ATENS)

## Abstract

**Background:**

Efflux pump activity has been associated with multidrug resistance phenotypes in bacteria, compromising the effectiveness of antimicrobial therapy. The development of methods for the early detection and quantification of drug transport across the bacterial cell wall is a tool essential to understand and overcome this type of drug resistance mechanism. This approach was developed to study the transport of the efflux pump substrate ethidium bromide (EtBr) across the cell envelope of *Escherichia coli *K-12 and derivatives, differing in the expression of their efflux systems.

**Results:**

EtBr transport across the cell envelope of *E. coli *K-12 and derivatives was analysed by a semi-automated fluorometric method. Accumulation and efflux of EtBr was studied under limiting energy supply (absence of glucose and low temperature) and in the presence and absence of the efflux pump inhibitor, chlorpromazine. The bulk fluorescence variations were also observed by single-cell flow cytometry analysis, revealing that once inside the cells, leakage of EtBr does not occur and that efflux is mediated by active transport. The importance of AcrAB-TolC, the main efflux system of *E. coli*, in the extrusion of EtBr was evidenced by comparing strains with different levels of AcrAB expression. An experimental model was developed to describe the transport kinetics in the three strains. The model integrates passive entry (influx) and active efflux of EtBr, and discriminates different degrees of efflux between the studied strains that vary in the activity of their efflux systems, as evident from the calculated efflux rates:  = 0.0173 ± 0.0057 min^-1^;  = 0.0106 ± 0.0033 min^-1^; and  = 0.0230 ± 0.0075 min^-1^.

**Conclusion:**

The combined use of a semi-automated fluorometric method and an experimental model allowed quantifying EtBr transport in *E. coli *strains that differ in their overall efflux activity. This methodology can be used for the early detection of differences in the drug efflux capacity in bacteria accounting for antibiotic resistance, as well as for expedite screening of new drug efflux inhibitors libraries and transport studies across the bacterial cell wall.

## Background

Efflux pumps are major defensive components of the bacterial cell wall that actively extrude noxious compounds from the periplasm and/or cytoplasm, thereby decreasing their intracellular concentration [1,2]. Active efflux of antibiotics by bacteria was first described in 1978 in *Escherichia coli *resistant to tetracycline [3] and, since then, it has been proven that the constitutive or inductive expression of these systems is responsible for the intrinsic and acquired resistance of many bacterial species to antimicrobials [4,5]. Bacterial efflux pumps may be divided into five categories, according to their bioenergetics and structural characteristics: the ATP-Binding Cassette (ABC) superfamily, the Major Facilitator Superfamily (MFS), the Resistance Nodulation cell Division (RND) family, the Small Multidrug Resistance (SMR) family and the Multidrug And Toxic compound Extrusion (MATE) family [5-7]. Efflux pumps may be specific for one substrate or recognize a wide range of structurally dissimilar and unrelated compounds, including several classes of antibiotics [7,8]. In *E. coli*, the most common commensal Gram-negative bacterium of the human gut, at least nine proton dependent efflux pumps responsible for multidrug resistant (MDR) phenotypes have been described [2,9-11]. Among these, the tripartite AcrAB-TolC system is recognized as the most important multi-component efflux system and is composed of: (i) AcrB, an inner membrane protein that functions as the transporter component of the pump; (ii) TolC, the protein that traverses the cell envelope and provides a conduit to the exterior; and (iii) AcrA, two periplasmic embedded membrane proteins that anchor TolC to the plasma membrane [12]. The AcrAB-TolC system is responsible for the extrusion of substrates from the periplasm and/or the cytoplasm. The over-expression of this efflux system is a major cause of the MDR phenotype of clinical isolates, due to its ability to extrude several classes of antibiotics [2,10,13]. Although AcrAB-TolC is considered the most important efflux system of *E. coli*, other tripartite efflux pumps, such as the AcrEF-TolC can extrude similar substrates albeit at lower efficiency levels. Moreover, these pumps can be over-expressed when AcrAB is deleted or inactivated [8,11,13,14].

Several methods have been used to detect and quantify the activity of bacterial efflux pump systems using radio-labeled, metal-labeled or fluorescent substrates to monitor their efflux in bacterial cells [15-21]. Bulk measurement techniques that use fluorescence spectroscopy yield a general understanding that represents the balance between entry and extrusion of a given substrate, which may result from the efflux activities of one or several pumps [17,19], and where cell membrane permeability also plays an important role [11,14]. Because the permeability to the substrate may be regulated by a decreased number of porins or by an increase of the lipopolysaccharide layer of the cell envelope [11,14], the demonstration of efflux activity invariably involves the use of an agent that promotes a significant increase of substrate accumulated [19,22,23]. Unless the demonstration of the role of a specific efflux pump is readily made, *i.e*. by the quantification of the expression level of a specific transporter [11,13], or by the use of mutants whose specific efflux pump has been deleted or inactivated [10], very little can be said about the role that any efflux pump system may play in the MDR phenotype of bacteria.

Recently, a semi-automated fluorometric method has been developed by us for the assessment of efflux pump activity in bulk bacterial cells [22]. The method uses the common efflux pump substrate ethidium bromide (EtBr) that has been shown to be a particularly suitable probe for these studies. In fact it emits weak fluorescence in aqueous solution (external to the cell) and becomes strongly fluorescent when its concentration within the periplasm exceeds that of the aqueous solution due to its binding to cellular components [16]. Such binding must be of a weak type if the agent is to serve as a useful probe for efflux, since later intercalation into DNA results in strong binding that precludes any dissociation of the substrate for efflux [16,17]. This method can distinguish accumulation, which reflects the balance between influx and efflux, from efflux itself [22] and, therefore, has the potential for the study of the kinetics of EtBr transport. The method was developed using the wild-type *E. coli *K-12 AG100 as the bacterial model and the accumulation and efflux of EtBr was monitored by real-time fluorometry using the Rotor-Gene™ 3000 from Corbett Research (Sydney, Australia) [22-24]. The major innovation of this method is the simultaneous evaluation of efflux pump activity *in vivo *and on-line of a large number of samples, with the possibility to expose the same cell preparation to different environmental conditions in a single assay.

In the study to be described herein, this method has been used to define and parameterize a mechanistic experimental model that demonstrates the kinetics of EtBr influx and efflux by *E. coli *strains that differ in their capacity to extrude this molecule. The determination of the influx and efflux rates (*k*^+ ^and *k*^-^, respectively) of a fluorescent substrate by the approach proposed allows the quantification of the cell overall efflux capacity. This information can be useful to interpret different phenotypes resulting from this efflux activity, including multidrug resistance in clinical bacterial strains.

## Methods

### Bacteria

The following bacteria were employed in this study: *E. coli *K-12 AG100 wild-type strain (*argE3 thi-l rpsL xyl mtl *Δ(*gal-uvrB*) *supE44*), containing a fully functional AcrAB-TolC efflux pump system; *E. coli *AG100A (Δ*acrAB*::Tn*903 *Kan^r^) with the AcrAB-TolC efflux pump system inactivated due to the insertion of the transposon Tn*903 *in the *acrAB *operon [10]; and *E. coli *AG100_TET_, a AG100 progeny strain, induced to high level of resistance to tetracycline (able to survive in 10 μg/ml of tetracycline, with an MIC of 12 μg/ml), as previously described, and over-expressing several efflux pumps, among which *acrAB *shows the highest expression level when exposed to high levels of tetracycline [11,13]. *E. coli *K-12 AG100 and AG100A have been previously characterized and were kindly offered by Hiroshi Nikaido (University of California, Berkeley, California, USA) [10]. Bacterial cultures were grown in Luria-Bertani (LB) medium at 37°C with agitation (220 rpm). Cultures of AG100A and AG100_TET _were supplemented with 100 μg/ml of kanamycin and 10 μg/ml of tetracycline, respectively.

### Reagents

Phosphate Buffered Solution (PBS), glucose, EtBr, kanamycin, ciprofloxacin, ofloxacin, chloramphenicol, erythromycin, tetracycline and chlorpromazine (CPZ) were purchased from Sigma-Aldrich Química SA (Madrid, Spain). EtBr solutions were stored at 4°C and protected from light. Tetracycline stock solution was prepared in methanol, whereas kanamycin, ciprofloxacin, ofloxacin, erythromycin and CPZ were prepared in distilled water and filtered with 0.22 μm syringe filters (Millipore Corporation, Bedford, USA). Chloramphenicol stock solution was prepared in ethanol. All working solutions were prepared in distilled water on the day of the experiment. LB medium was purchased from Difco (Detroit, Michigan, USA). Mueller-Hinton (MH) broth medium was purchased from Oxoid (Basingstoke, Hampshire, UK).

### Determination of minimum inhibitory concentrations (MICs)

The MICs for EtBr, the efflux pump inhibitor (EPI) CPZ, kanamycin, ciprofloxacin, ofloxacin, chloramphenicol, erythromycin and tetracycline were determined by the broth microdilution method in 96-well microtitre plates according to the CLSI guidelines [25]. Briefly, bacterial strains were incubated overnight in 5 ml of MH broth at 37°C with shaking at 220 rpm. Bacterial cultures were then diluted in PBS to a McFarland 0.5 turbidity standard. Aliquots of 0.05 ml were transferred to each well of the 96-well plate that contained 0.15 ml of each compound at concentrations prepared at 2-fold serial dilutions in MH broth medium. The plates were incubated at 37°C and the MIC results registered after 16-18 h. The MIC was defined as the lowest concentration of compound for which no growth was observed. In order to assure that the EPI did not compromise the cellular viability, the concentrations used in the following work did not exceed 1/2 of the MIC. The MIC for tetracycline was also calculated in the presence of CPZ at 1/2 of the MIC.

### The semi-automated fluorometric method

**Figure 1:**
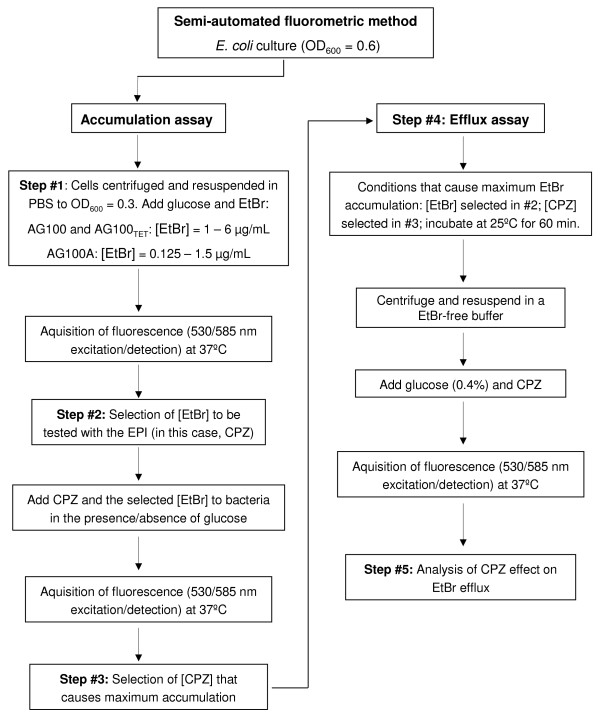
**Experimental flowchart of the semi-automated fluorometric method**.

The method was carried out using the real-time thermocycler Rotor-Gene™ 3000 (Corbett Research, Sidney, Australia) to monitor the accumulation and extrusion of EtBr on a real-time basis [22,23]. The instrument allows the selection of the excitation and emission wavelengths that for EtBr are 530 nm band-pass and 585 nm high-pass filters, respectively. The fluorescence was acquired in cycles of 60 seconds, during the desired time interval, at the required temperature (37°C or 25°C). An explanatory diagram is provided in Figure 1.

#### (i) Accumulation assay

The accumulation of EtBr was carried out as previously described [22]. Briefly, the *E. coli *strains were grown in 10 ml of LB medium until they reached a mid-log phase, which corresponded to an optical density at 600 nm (OD_600_) of 0.6. The bacteria were then centrifuged at 16,060 ×*g *for 3 minutes, the pellet washed twice with the same volume of PBS and the OD_600 _of the cellular suspension adjusted to 0.3. Glucose was added to the cellular suspension to a final concentration of 0.4% (v/v) and aliquots of 0.095 ml were transferred to 0.2 ml microtubes. EtBr was added in aliquots of 0.005 ml to obtain final concentrations that ranged from 1.0 to 6.0 μg/ml for *E. coli *K-12 AG100 and AG100_TET _and from 0.125 to 1.5 μg/ml for AG100A. The fluorescence was monitored in the Rotor-Gene™ 3000 at 37°C under the conditions described above. The effect of CPZ in the accumulation of EtBr was determined under conditions that optimize efflux (presence of glucose and incubation at 37°C). CPZ was used at concentrations that did not exceed 1/2 the MIC and the fluorescence was measured over a period of 60 minutes.

#### (ii) Efflux assay

The assessment of the efflux of EtBr was conducted as previously described [22]. Briefly, the *E. coli *strains were loaded with EtBr under conditions that favor accumulation (no glucose, 25°C and presence of CPZ). When the maximum level of EtBr accumulation was reached (approximately 60 minutes), the bacteria were centrifuged (16,060 ×*g *for 3 minutes) and the medium was replaced by: i) PBS without glucose; ii) PBS containing glucose; and iii) PBS without glucose and with CPZ (control of minimum efflux). Aliquots of 0.1 ml were transferred to 0.2 ml microtubes and the assay was performed at 37°C with continuous measurement of fluorescence as described above. The efflux of EtBr is presented in terms of relative fluorescence, which is obtained from the comparison between the fluorescence observed for the bacteria in the presence or absence of glucose and the control in which the cells are exposed to conditions of minimum efflux (*i.e*., absence of glucose and presence of CPZ). Each experiment was conducted in triplicate and the results obtained did not vary.

### Model to quantify EtBr efflux in *E. coli*

#### (i) Quantification of intracellular EtBr concentration

In order to assess the amount of EtBr extruded from the cells, one has to quantify the amount retained inside the cells. First, a calibration curve is determined that correlates the initial fluorescence of the buffer containing EtBr (FL_o_) with the corresponding EtBr concentration (varying from 0 to 6 μg/ml): [EtBr]_*intial *_= 0.1201 × FL_0 _+ 0.0373(R = 0.99). Then, for each concentration tested, an EtBr accumulation assay was performed, as described above. To quantify the intracellular EtBr concentration, at the end of the accumulation assay, 0.6 ml of each EtBr containing buffer were separated from the cells by centrifugation at 16,060 ×*g *for 10 minutes and the supernatant filtered with 0.22 μm syringe filters from Millipore Corporation (Bedford, USA). The fluorescence of the cell free buffer was measured in the Rotor-Gene™ 3000 (FL(PBS+EtBr)_filt non corrected_).

Since some EtBr is retained by the filter itself, a correction factor must be used to account for this difference. Thus, the corrected fluorescence of the filtered buffer corresponds to (FL(PBS+EtBr)_filt corrected_). = 1.2289 × (FL(PBS+EtBr)_filt non corrected_). + 0.7032 (R = 0.99). The difference between the initial fluorescence of the EtBr containing buffer (FL_o_) and (FL(PBS+EtBr)_filt corrected _gives the fluorescence corresponding to the intracellular EtBr concentration. However, the fluorescence data from the transport assays measured experimentally still contains a component from the EtBr that remains in solution. Thus, each value of the experimental accumulation curve can then be corrected by subtracting the background corresponding to the fluorescence of the initial EtBr concentration, originating FL_int exp corr_. By plotting the previously determined EtBr intracellular concentration for the final value of FL_int exp corr_, for each initial EtBr concentration tested, a new relation is achieved: [EtBr]_int _= 0.022 × FL_int exp corr_+ 0.06 (R = 0.99), which allows the conversion of the experimental fluorescence data over time into intracellular EtBr concentration over time. The correlations shown here were determined for the wild-type *E. coli *K-12 AG100. Similar relations were determined specifically for the other two strains used (AG100A and AG100_TET_).

#### (ii) Mathematical and statistical analyses

The model was fitted to the experimental values ([EtBr]_int _*vs t*) using the software *Table Curve*™ 2D from Jandel Scientific - AISN Software STATISTICA, which allowed the determination of the values of the parameters describing the influx and efflux rates, *k*^+ ^and *k*^-^, respectively. Three independent assays were conducted and the results shown are the average ± standard deviation. P-value from the ANOVA statistical test was calculated.

#### (iii) Model validation

The predictive model proposed was validated by comparing the experimental data obtained using *E. coli *K-12 AG100, AG100A and AG100_TET _cell suspensions incubated in the presence of different concentrations of EtBr and the curves generated by the model, using the *k*^+ ^and *k*^- ^constants determined as described above.

### Flow cytometry

This methodology was used to corroborate the analysis of the data obtained with the semi-automated fluorometric method. Therefore, the samples from the accumulation and efflux assays, used in the semi-automated fluorometric method, were also analyzed by flow cytometry, in order to compare the results obtained with these two methodologies. Data acquisition and analysis were performed using a FACSCalibur™ (BD Biosciences, San Jose, CA, USA). EtBr was excited at 488 nm and the fluorescence detected through a 585 nm filter (FL-2 channel).

#### (i) Accumulation assay

*E. coli *strains were cultured in 10 ml of LB medium at 37°C and 220 rpm until an OD_600 _of 0.6. Aliquots of 1.0 ml were centrifuged at 16,060 ×*g *for 3 minutes, the supernatant discarded and the pellet washed twice with PBS. The OD_600 _of the bacterial suspension was adjusted to 0.3 using PBS without glucose. EtBr was added at a final concentration of 1 μg/ml and CPZ was added to a final concentration of 20 μg/ml. Following incubation at 25°C for 60 minutes, aliquots of 0.5 ml were taken for fluorescence measurement in the flow cytometer FACSCalibur™. Data was collected for at least 10,000 events per sample.

#### (ii) Efflux assay

After loading the bacteria with EtBr (1 μg/ml) in the presence of CPZ (20 μg/ml), the bacterial suspension was centrifuged at 16,060 ×*g *for 3 minutes. The supernatant was removed and the pellet resuspended in EtBr-free PBS, adjusting the OD_600 _to 0.3. Efflux was assessed in the presence and absence of glucose at 0.4% (v/v). Aliquots of 0.5 ml were taken after 2.5, 5, 15, 30 and 60 minutes after incubation at 37°C, for fluorescence measurement in the flow cytometer FACSCalibur™. Analyses were performed with an acquisition of at least 10,000 events per sample.

## Results and Discussion

### Detecting EtBr efflux mediated by *E. coli *efflux pump systems

**Figure 2:**
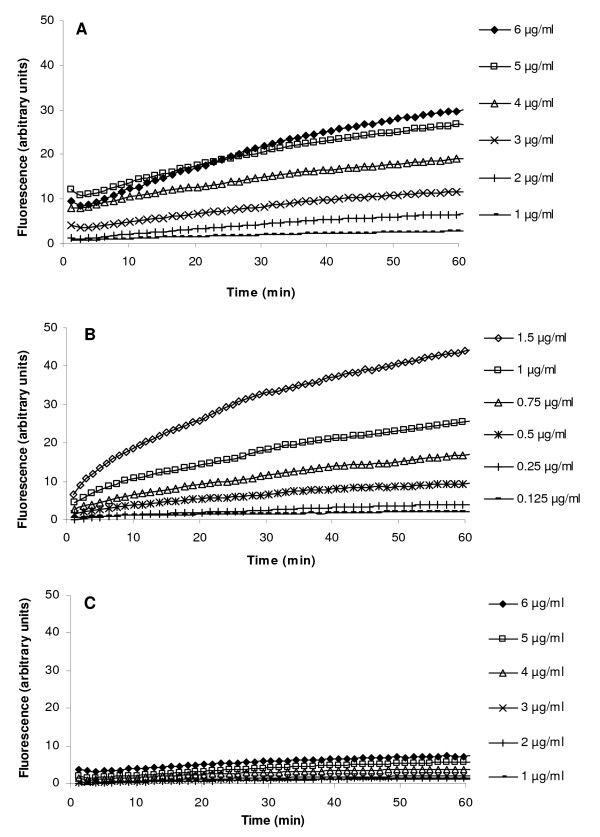
**Accumulation of EtBr at increasing concentrations by *E. coli *K-12 AG100 (A), AG100A (B) and AG100_TET _(C)**. The bacteria were exposed to increasing concentrations of EtBr at 37°C in the presence of glucose. **Note**: assays for AG100A were carried out with lowest EtBr concentrations (0.125 μg/ml - 1.5 μg/ml) -- see text.

Experiments were carried out with EtBr concentrations that did not affect in any way the cell viability, *i.e*., that did not exceed 1/2 the MIC for EtBr (Table 1). In this case and as previously demonstrated, accumulation of EtBr and its extrusion from *E. coli *are the result of the balance between EtBr entry by passive diffusion (influx) and the extrusion activity of efflux pump systems, primarily the AcrAB-TolC [11,13]. As shown by Figure 2, the accumulation of EtBr under conditions that are considered to maximize efflux, *i.e*., presence of glucose and 37°C, begins to take place at a concentration of EtBr that exceeds 1.0 μg/ml for the wild-type *E. coli *K-12 AG100 (Figure 2A), 0.250 μg/ml for the AG100A (*acrAB *inactivated; Figure 2B), and 1.0 μg/ml for the AG100_TET_(*acrAB *and other efflux pump genes over-expressed; Figure 2C). As expected, the strain that over-express efflux systems (AG100_TET_) accumulates the least amount of EtBr even when the maximum concentration of EtBr to which it is exposed is 6 μg/ml. On the contrary, the AG100A, whose AcrAB efflux pump has been inactivated, accumulates approximately 25 times more EtBr when exposed to a far lower concentration of 1.5 μg/ml of EtBr. The importance of the AcrAB-TolC efflux system is further illustrated by the comparison of accumulation of EtBr by the wild-type AG100 to that by the AG100A, when both strains are exposed to 1 μg/ml of EtBr.

**Table 1:** MIC values of EtBr and several antibiotics for *E. coli *AG100, AG100A and AG100_TET_.

	**MIC (μg/ml)**							
***E. coli *strains**	**EtBr**	**KAN**	**CIP**	**OFX**	**CHL**	**ERY**	**TET**	**TET + CPZ**

AG100	150	15	0.03	0.12	8	100	2.0	0.5
AG100A	5	> 200	0.004	0.015	2	6.25	0.5	0.5
AG100_TET_	300	10	0.12	0.48	> 16	100	12	3

Ethidium Bromide (EtBr); Kanamycin (KAN); Ciprofloxacin (CIP); Ofloxacin (OFX); Chloramphenicol (CHL); Erythromycin (ERY); Tetracycline (TET); and Chlorpromazine (CPZ). Kanamycin was included as control for the presence of Tn*903 *in *E. coli *AG100A (Δ*acrAB*::Tn*903 *Kan^r^). The tetracycline MICs were also calculated in the presence of 30 μg/ml (AG100), 10 μg/ml (AG100A) and 70 μg/ml (AG100_TET_) of CPZ (1/2 CPZ MIC).

**Figure 3:**
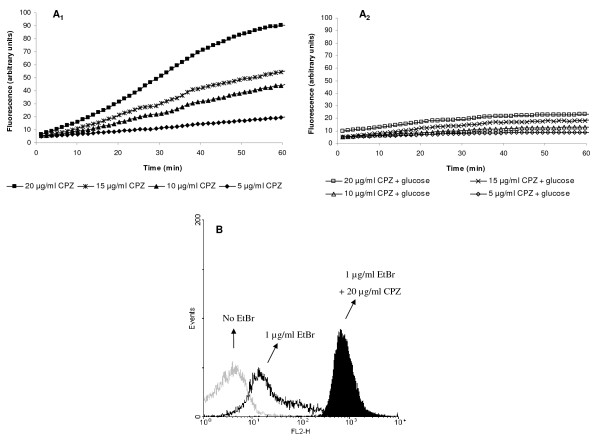
**Effect of chlorpromazine (CPZ) on EtBr accumulation by *E. coli *K-12 AG100**. The bacteria were loaded with EtBr at 1 μg/ml in the presence of increasing concentrations of CPZ for a period of 60 minutes at 37°C in the presence and absence of glucose and accumulation assessed by: (A1 and A2) the semi-automated fluorometric method; (B) flow cytometry, represented by an histogram overlay corresponding to the data selected for analysis (2^nd ^gate) for *E. coli *K-12 AG100 (i) without EtBr or CPZ, (ii) after 60 minutes of exposure to EtBr (1 μg/ml) and (iii) after 60 minutes of exposure to EtBr (1 μg/ml) and CPZ (20 μg/ml) at 37°C without glucose. Data in graphics A1 and A2 correspond to the same assay and were separated for the sake of figure clarity.

Accumulation of EtBr inside the bacterial cells can be increased in the presence of an EPI such as phenothiazines (*e.g*. CPZ), compounds that have been shown to inhibit efflux activity of Gram-negative bacteria [4,5,22]. Thus, these compounds are usually employed for the demonstration of over-expressed efflux pumps of bacteria [5,19,22,23]. Because of the real-time capacity and sensitivity of the semi-automated fluorometric method, the demonstration of the effects of an EPI, such as CPZ, on the intrinsic efflux system of the wild-type *E. coli *K-12 AG100 is readily made. As shown by Figure 3A (A1 and A2), high concentrations of CPZ maximize the amount of EtBr accumulated only when glucose is absent from the medium. Similar results were obtained for the strains AG100_TET _and AG100A (data not shown). The effect of CPZ on the accumulation of EtBr by wild-type *E. coli *K-12 AG100 is similarly demonstrated and confirmed with the aid of flow cytometry (Figure 3B).

**Figure 4:**
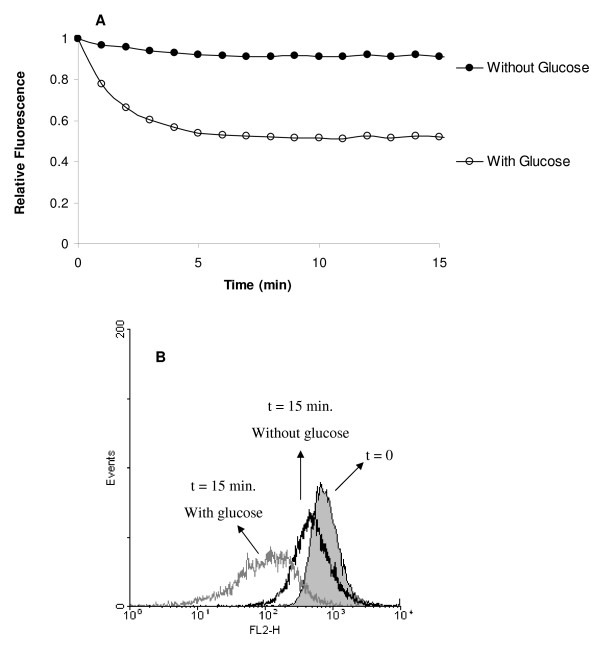
**Detection of EtBr efflux in *E. coli *K-12 AG100**. The bacteria were loaded with EtBr at 1 μg/ml in the presence of CPZ (20 μg/ml) for a period of 60 minutes at 25°C. After replacing the buffer with EtBr-free PBS with or without glucose, the efflux was assessed by: (A) the semi-automated fluorometric method, performed at 37°C for 15 minutes; and (B) flow cytometry, represented by an histogram overlay corresponding to the data selected for analysis (2^nd ^gate) for *E. coli *K-12 AG100 immediately after replacing the buffer with EtBr-free PBS (t = 0) and 15 minutes after incubation at 37°C with or without glucose.

The on-line visualization of efflux activity by the EtBr loaded cells requires that accumulation of EtBr has previously taken place. Therefore, before performing the efflux assays, bacteria are exposed to conditions that promote significant accumulation of EtBr [22,23]. To this extent, we have chosen a temperature of 25°C, absence of glucose and presence of CPZ (20 μg/ml), to promote maximum accumulation, within 60 minutes. When maximum accumulation has taken place under these conditions, the bacteria are washed free of EtBr and CPZ, and resuspended in fresh buffer with and without glucose. Fluorescence readings were conducted over a period of 15 minutes at 37°C. As shown by Figure 4, restoration of optimum conditions of glucose, noted to prevent accumulation, are required for the extrusion of EtBr. This assay shows that *E. coli *cells need an energy source for efflux to take place.

Therefore, this methodological approach proved its usefulness for the demonstration of active efflux of EtBr in *E. coli *and the strategy developed allowed to differentiate influx (passive diffusion into the cell) from efflux activity (active efflux through pumps).

### Modeling EtBr transport across *E. coli *cell wall

In order to quantify the efflux activity, a mechanistic experimental model that establishes a balance related to the intracellular EtBr concentration was developed, as described below:

(1.1)

where [EtBr]_int _is the intracellular EtBr concentration (μg/ml); [EtBr]_ext _is the external EtBr concentration (μg/ml); k^+ ^is the rate of EtBr entry into the cells (min^-1^) and k^- ^corresponds to the rate of EtBr efflux from the cells (min^-1^). Therefore,

(1.2)

where [EtBr]_Tot _is the total EtBr concentration (μg/ml). Replacing equation (1.2) in equation (1.1) and solving it for [EtBr]_int_, it is possible to obtain the following equation, which describes the EtBr transport across the cell wall of *E. coli*:

(1.3)

The equation developed herein (eq. 1.3) allied to the technical approach used to quantify the intracellular EtBr concentration (Methods) allowed us to quantify the degree of EtBr efflux activity in the three *E. coli *strains (AG100, AG100A and AG100_TET_). This strategy is based on the distinct levels of EtBr fluorescence in solution and inside the cells. EtBr emits fluorescence when it is free in solution (in this case, PBS) in a concentration dependent manner, accurately captured by the photomultiplier detector with the 585 nm high-pass filter of the Rotor-Gene™ 3000 that differentiates μg/ml differences [22,24]. The high sensitivity of this detector ensue the definition of a calibration curve, which correlates fluorescence (FL_0_) and the initial EtBr concentration in solution, before the cells are added to the medium, varying from 0 μg/ml to 6 μg/ml [24]:



where [EtBr]_initial = _[EtBr]_Total_. When the *E. coli *cells are exposed to EtBr containing buffer (1 to 6 μg/ml for AG100 and AG100_TET_; 0.125 to 1.5 μg/ml for AG100A), the cells start to accumulate EtBr that enters by passive diffusion, with the efflux pump systems trying to balance this entry according to the kinetics presented in Figure 2. At these low concentrations, EtBr does not reach the cellular components in the cytoplasm to which it could bind irreversibly, being effluxed from the cytoplasm and periplasm by either single- or multi-component efflux systems [26,27]. When the concentration of EtBr within the periplasmic space exceeds significantly its extrusion, EtBr is translocated beyond the inner plasma membrane where the medial sites of the cell contain nucleic acids to which the EtBr can intercalate almost irreversibly, after which extrusion is not possible [16,17,26,27].

**Figure 5:**
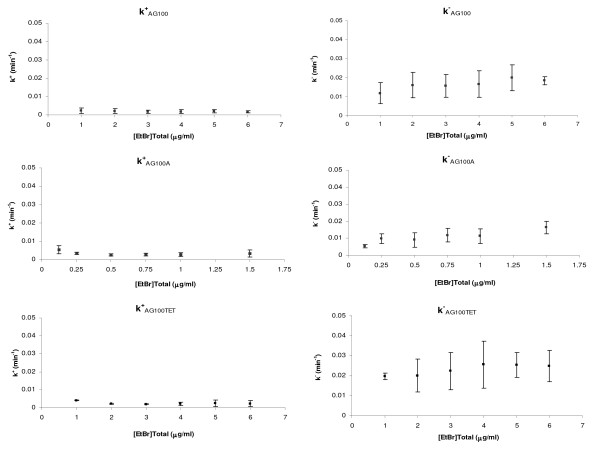
**Influx (k^+^) and efflux (k^-^) rates of EtBr in the *E. coli *strains tested**. The values shown represent averages of three independent experiments and respective standard deviations for the three *E. coli *strains tested: AG100, AG100A and AG100_TET_.

Using the rationale described in Methods, it is possible to correlate the fluorescence readings by the semi-automated fluorometric method in bulk cells to the EtBr concentration that remains inside the cells; [EtBr]_int _= 0.022 × FL_int exp corr _+ 0.06 (R = 0.99). With the assistance of the software *Table Curve*™ 2D from Jandel Scientific - AISN Software STATISTICA, it is then possible to adjust the model (eq. 1.3.) to the experimental data and determine the values of the constants k^+ ^and k^-^, which represent a measure of the rate of entry and the rate of efflux of EtBr into and out of the cell, respectively (Figure 5). Table 2 summarizes the values of k^+ ^and k^- ^obtained for the three strains tested. The values obtained show similar influx rates for the three *E. coli *strains studied, as expected, since this parameter reflects the entry of EtBr by passive diffusion. Both the passive influx absolute values and their variation among the three strains are negligible when compared to the values obtained for the efflux rates.

**Table 2:** Influx (k^+^) and efflux (k^-^) rates for *E. coli *K-12 AG100, AG100A and AG100_TET_.

***E. coli *strains**	**k^+ ^(min^-1^)**	**k^- ^(min^-1^)**
AG100	0.0019 ± 0.0009	0.0173 ± 0.0057
AG100A	0.0035 ± 0.0012	0.0106 ± 0.0033
AG100_TET_	0.0025 ± 0.0009	0.0230 ± 0.0075

k^+ ^and k^- ^were determined using the software Table Curve™ 2D from Jandel Scientific - AISN Software STATISTICA to adjust the model (eq. 1.3.) to the experimental data obtained with the semi-automated fluorometric method (Figure 2). The results represent an average of three independent assays with the correspondent standard deviation.

The model proposed accurately describes the behavior of the three strains, showing a lower efflux rate for the AG100A strain when compared to the wild-type AG100 (P value = 0.0023), as a result of the inactivation of the AcrAB-TolC system in the mutant strain. For the AG100_TET _strain the model revealed a significantly increased efflux rate compared to the wild-type strain (P value = 0.0057). This result reflects the higher efflux activity in this tetracycline adapted strain due to the over-expression of efflux pumps, particularly of the AcrAB-TolC system [11,13].

Moreover, the results provided by the model, in terms of EtBr efflux rates, correlate with the MIC for EtBr for each of the strains studied (Table 1). While the wild-type strain, *E. coli *AG100 (MIC_EtBr _= 150 μg/ml), showed a EtBr efflux rate of  = 0.0173 ± 0.0057 min^-1^, the AcrAB deleted strain, *E. coli *AG100A (MIC_EtBr _= 5 μg/ml), had the lowest EtBr efflux rate  = 0.0106 ± 0.0033 min^-1^. Conversely, the tetracycline induced strain, *E. coli *AG100_TET_, over-expressing the AcrAB system, shows a much higher efflux rate  = 0.0230 ± 0.0075 min^-1^, which corresponds to an MIC for EtBr of 300 μg/ml. MICs of each strain towards several antibiotics known to be efflux pump substrates also showed a correlation between efflux activity and resistance level to these drugs (Table 1). The determination of the MICs for tetracycline in the presence of the EPI CPZ reinforced the role played by efflux on such resistance, as the MICs values decreased in the presence of sub-inhibitory concentrations of this EPI, according to the efflux activity previously determined for each strain (Table 1).

**Figure 6:**
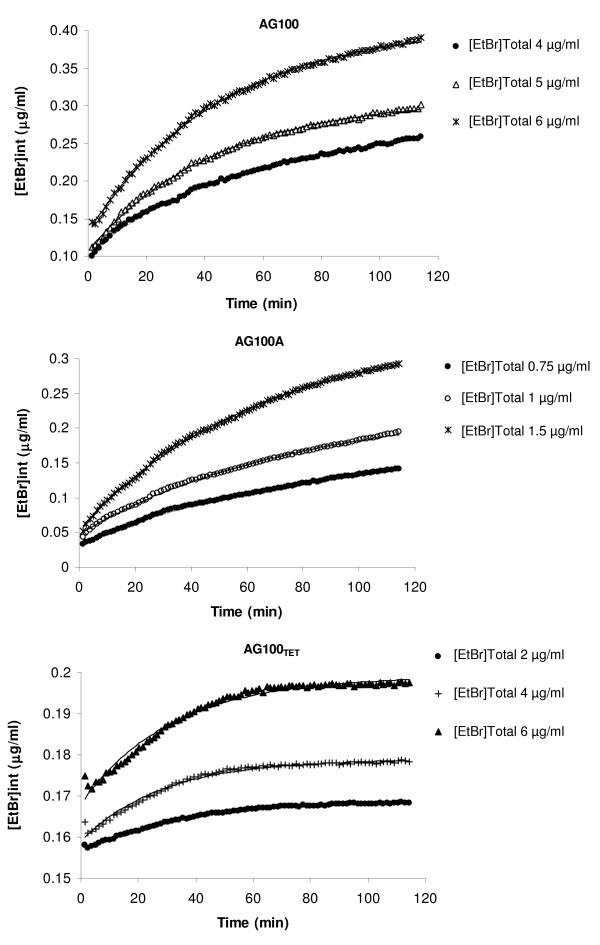
**Examples of model fitting to experimental data**. The figures represent the fitting of the model to the experimental data for the time course of intracellular EtBr concentration in *E. coli *strains AG100, AG100A and AG100_TET_, incubated in the presence of different EtBr concentrations. In each case, the solid line represents the model fit.

This approach allowed us, for the first time in the literature, to calculate the values of EtBr influx/efflux rates (k^+ ^and k^-^) for *E. coli *and to construct a predictive model for the accumulation curve of this molecule at any concentration by a given *E. coli *strain, provided that EtBr concentration does not affect the cellular viability. An example of such calculation is provided in Figure 6, for three different concentrations of EtBr for the *E. coli *strains used. When these predictive curves are compared to the experimental data a close fit is obtained (Figure 6), thus validating the proposed model.

## Conclusion

In this work we have applied the technical advantages of the semi-automated fluorometric method [22] for the development and application of an experimental model to describe and quantify the efflux pump activity in *E. coli*. The semi-automated fluorometric method affords the *in vivo *and on-line evaluation of the efflux pump activity by real-time fluorometry using the Rotor-Gene™ 3000, and represents an innovative technique when compared to other technologies [23]. Based upon the differences of fluorescence that reflect differences on the EtBr concentrations outside and inside the cells, the technique provides a powerful tool for the real-time monitoring of efflux kinetics in bacterial cells. Moreover, it has potential for the identification of new efflux pump substrates, as well as new bacterial EPIs [23]. A more fundamental application is the extension of this methodology to understand the transport kinetics of EtBr or other fluorescent substrates in bacterial cells.

We used such approach to differentiate and quantify EtBr transport in *E. coli*, and an experimental model was developed which parameterizes passive entry (influx) and active efflux of EtBr. The methodology developed allowed to detect differences in the EtBr efflux activity among strains that differ in their efflux pump expression, highlighting the importance of these systems in the extrusion from the cell of toxic compounds such as EtBr [6,7,10].

Although the experimental model presented was designed based upon a set of three canonic, isogenic strains, as a way to assess the method's sensitivity to differentiate their singular efflux activities, it can be applied to other *E. coli *strains or bacteria, since it is based on the overall transport of EtBr across the bacterial cell wall, which is common among bacteria [2,7,16,22,23]. It should be stressed however that it allows measuring the overall efflux activity rather than efflux related to a specific pump. For evaluation of the role played by a pump, a mutant specific for that pump must be used. The determination of influx and efflux rates for each of the strains to be analyzed opens new insights and opportunities for exploration of cell wall permeability, as well as for the control of the efflux activity in bacterial cells, representing a quantitative method to determine efflux activity. This approach may be used to elaborate a standardized data base for different reference strains relative to efflux activity (efflux rates), providing the means by which one can evaluate the resistance due to efflux pump activity in standardized conditions between laboratories. It represents a particularly suitable procedure for the assessment of efflux pump activity in MDR clinical isolates and screening for potential inhibitors of such activity. Such approaches may contribute to a better understanding of the bacterial efflux systems as a resistance mechanism and to design new therapeutic strategies against MDR bacterial infections [28].

## Competing interests

The authors declare that they have no competing interests.

## Authors' contributions

LP and LR carried out the experimental work related to accumulation and efflux assays and MIC determination. LP and IC participated in the development of the model and LR assisted in the flow cytometry experiments. LP, LR and IC assisted in the preparation of the manuscript. MM carried out the flow cytometry experiments. PF and CCCRC participated in the development and validation of the experimental model and assisted in the preparation of the manuscript. GAM and LA assisted in the design of the study. LA assisted in the preparation of the manuscript. FS assisted in the design and performance of the flow cytometry experiments. MV conceived the study, its design and coordination and prepared the draft of the manuscript.

All authors read and approved the final manuscript.
